# Jitter and muscle fiber conduction velocity in long COVID fatigue

**DOI:** 10.1055/s-0045-1802961

**Published:** 2025-02-24

**Authors:** João Aris Kouyoumdjian, Leticia Akemi Rama Yamamoto, Carla Renata Graca

**Affiliations:** 1 Faculdade Estadual de Medicina de São José do Rio Preto (FAMERP), Departamento de Ciências Neurológicas, Psiquiatria e Psicologia Médica, Laboratório de Investigação Neuromuscular, São José do Rio Preto SP, Brazil.

**Keywords:** SARS-CoV-2, Post-Acute COVID-19 Syndrome, Fatigue, Single-Fiber Electromyography, Muscle Fiber Conduction Velocity

## Abstract

**Background** Long coronavirus disease (long COVID, LC) is defined as the continuation or development of new symptoms 3 months after the acute stage of severe acute respiratory syndrome coronavirus 2 (SARS-CoV-2) infection. In LC, the rate of fatigue/postexertional malaise (F-PEM) has been described to be as high as 70%, regardless of age or severity of the acute symptoms.

**Objective** To evaluate the neuromuscular junction (NMJ) function and the isolated muscle fiber conduction velocity (MFCV) in situ in LC cases and controls.

**Methods** We studied 37 subjects without SARS-CoV-2 (controls) and 32 cases of SARS-CoV-2 infection, half with LC symptoms (LC-yes) and half without them (LC-no). Single-fiber electromyography (jitter measured with a concentric electrode), MFCV, the fast-to-slow MFCV ratio (F/S ratio), and the motor unit potentials (MUPs) were taken in the tibialis anterior muscle.

**Results** At least 1 jitter parameter was abnormal in 1/37 controls, in 1/16 LC-no patients, and in 2/16 LC-yes patients, without significant differences among them. None of the subjects with abnormal jitter presented fluctuation symptoms or positive acetylcholine-receptor antibody. The MFCV and F/S ratios did not show abnormalities in any of the participants. The MUPs did not show myopathic or neurogenic abnormality in needle electromyography. The most frequent symptom in LC was F-PEM, which occurred in all LC-yes patients and was significantly different from the other groups.

**Conclusion** Fatigue/postexertional malaise was found in all cases of LC, and the electrophysiological findings did not indicate the muscle fiber or the NMJ as a relevant factor in this condition.

## INTRODUCTION

In December 2019, unusual cases of pneumonia were reported in China, which quickly spread to other parts of the country and then worldwide. This outbreak was confirmed to be caused by a novel coronavirus (CoV). 1 The symptomatology was similar to that of severe acute respiratory syndrome coronavirus 1 (SARS-CoV-1) in 2003; thus, the virus was termed SARS-CoV-2. Since then, the World Health Organization (WHO) has counted 775,583,309 cases, with 7,050,691 deaths. 2 These numbers are likely much higher due to many undocumented cases. 3

The WHO defines long coronavirus disease (long COVID, LC) as the continuation or development, without explanation, of new symptoms 3 months after the initial SARS-CoV-2 infection. 4 5 Long COVID can manifest in approximately 20% of SARS-CoV-2 infections, and the most frequent symptom is fatigue/postexertional malaise (F-PEM), which has been described in as much as 70% of the cases of SARS-CoV-2 infection. 6 7 8 9 10 It can affect anyone exposed to SARS-CoV-2, regardless of age or the severity of the acute symptoms. 11

Single-fiber electromyography (SFEMG) tests the function of the neuromuscular junction (NMJ) by measuring the jitter. 12 The symptoms of most junctional disorders typically include fluctuating weakness that initially could resemble fatigue.

Muscle fiber conduction velocity (MFCV) in situ was described in detail by Stålberg, 13 who found an average value of 3.70 m/s. The MFCV correlates directly with the gauge of muscle fibers and, in rare cases, with muscle membrane inexcitability.

The present study aims to evaluate the NMJ function by measuring the jitter and the muscle fiber function by measuring the MFCV in patients with a previous acute SARS-CoV-2 infection that subsequently present LC or not. In parallel, subjects without acute SARS-CoV-2 infection were studied as the control group.

## METHODS

### Study sample

The current study was conducted in accordance with the 1975 Declaration of Helsinki, and it was approved by the Ethics Committee of Faculdade de Medicina de São José do Rio Preto, in the state of São Paulo, Brazil (under number 4904517), where the tests were conducted. All subjects signed the informed consent form.

In total, 37 controls and 32 patients, adults of both sexes, were invited to participate from July 2021 to November 2023. They were randomly selected from our medical school and teaching hospital. The 32 patients with previous SARS-CoV-2 infection were split into 2 groups equally distributed by sex. The cases in the LC-no group did not present any symptoms after the acute infection, and the cases in the LC-yes group presented persisting or new symptoms after 6 months.

The inclusion criteria for the controls were good health and no previous SARS-CoV-2 infection. The inclusion criteria for the patients were symptomatic SARS-CoV-2 infection with a positive polymerase chain reaction (PCR) test or a few with the presence of antibodies. The exclusion criteria for all subjects and patients were: a dmission to the intensive care unit (ICU) for ventilatory support; previous, hereditary, or acquired neuromuscular diseases; comorbidities that can cause chronic peripheral neuropathy, such as diabetes mellitus or alcohol abuse; botulinum neurotoxin (BoNT) injection in the lower limbs; previous injuries to the lower limbs; and previous surgery on the lumbosacral spine.

The interview and the neurophysiological tests took about 3 hours for each participant. Firstly, a simple question was asked to select cases for the LC group: “Did something change in your life after the SARS-CoV-2 infection?” The assessment of F-PEM was made by adding the points assigned to the answers to the following questions:

- Do you feel disproportionately tired after minimal-intensity exercise?;
- Do you feel exhaustion after a light activity?;
- Do you feel pain after non-strenuous activities?;
- Do you feel “dead tired” after exercise?; and
- Do you feel mentally tired after minor activity?

The points for each question were assigned as follows: 0 for “no”, 1 for “sometimes”, 2 for “about half of the time”, 3 for “most of the time”, and 4 for “continuous”.

### Clinical summary

During the interview, the patients were asked about 13 symptoms of acute SARS-CoV-2 infection: fever, cough, sore throat, rhinorrhea, dyspnea, expectoration, myalgia, tiredness, fatigue, headache, diarrhea, olfaction loss, and gustation loss. We took the yes or no answers for each and split them into two categories: ≤ 5 and ≥ 6 affirmative answers.

### Electromyography

**Figure 1:**
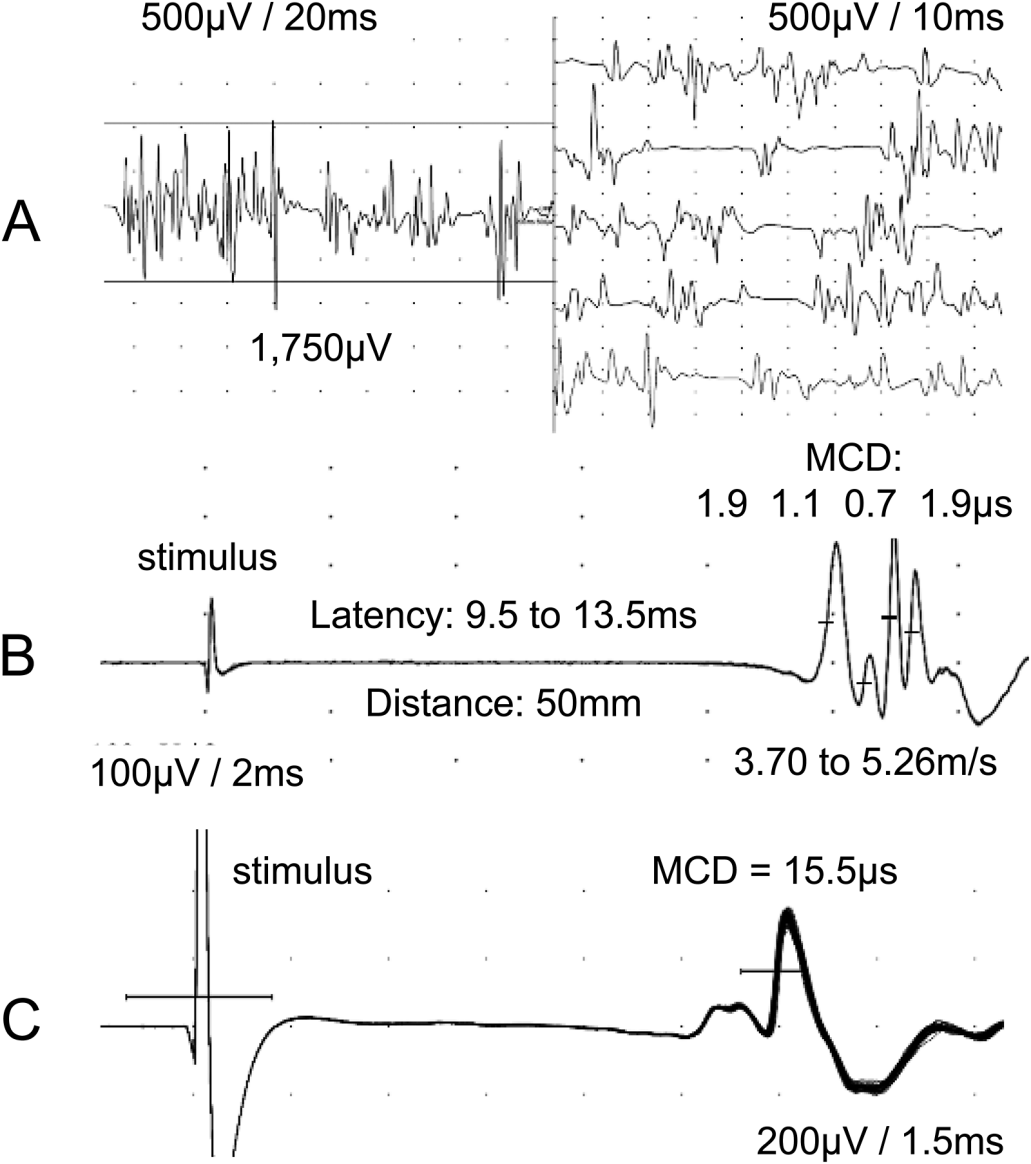
( **A** ) Motor unit potentials from the tibialis anterior muscle in semiquantitative electromyography, showing a mean amplitude of 1,750 µV. ( **B** ) Muscle fiber conduction velocity in situ, showing a complex of isolated muscle fiber action potentials with a conduction velocity varying from 3.70 m/s to 5.26 m/s. The mean consecutive differences were displayed above each spike and were shorter than 5 µs, defining direct isolated muscle fiber stimulation. ( **C** ) Isolated muscle fiber action potential after electrical activation, showing a mean consecutive difference (jitter) of 15.1 µs, defining indirect muscle fiber stimulation.

The first author (JAK) performed the needle electromyography (EMG) in the tibialis anterior (TA) muscle before the SFEMG and the MFCV tests. A machine with UltraProTM S100 Elite software in Synergy mode (Natus Neurology Incorporated, Middleton, WI, United States) was used for all participants. We analyzed it at rest for the presence of fibrillation or positive wave potentials and with a slight voluntary effort for the MUP recording. The MUP analysis was performed after a 20-second video recording. The MUPs could be analyzed in edition mode, changing the sweep and amplitude. Horizontal and vertical lines were used to measure the MUP duration (ms) and amplitude (µV) ( Figure 1A ).

### Single-fiber electromyography

The SFEMG was performed by the first author (JAK) with the same machine used for the needle EMG. Our previous article 14 detailed the electrical activation technique (stimulation) to measure the jitter in the TA muscle and defined the reference values. In short, the recordings were performed using a disposable concentric needle electrode (CNE; 25 mm x 30 G, Dantec DCN, Natus Neurology Incorporated). The electrical stimulation was performed using an intramuscular monopolar needle electrode (25 × 0.36 mm, 28G, Neuroline, Ambu A/S, Ballerup, Denmark). Stimulation at 10 Hz was delivered as rectangular pulses lasting 0.05 ms; the stimulus intensity was adjusted to produce a slight muscle twitch (1–2 mA). A CNE was inserted into the muscle's twitching part and gently positioned to record clearly-defined single-fiber action potentials (SFAPs) or apparent-SFAPs (aSFAPs), referred to as spikes. The stimulation intensity was finely adjusted to avoid submaximal stimulation and false increased jitter. Acceptable spikes should have a fast-rising phase (< 300 μs) without notches or shoulders, the shape should be constant at consecutive discharges, and the negative-going deflection in the waveforms should be parallel on superimposed traces 15 ( Figure 1C ). The low-frequency filter was set to 1,000 Hz to suppress distant muscle fiber activity. 12 The jitter was expressed as the mean consecutive difference (MCD) from 50 to 100 spikes and measured by the amplitude (level), except for riding spikes, where the peak was used for measurement. For each participant, 30 MCD values were obtained. In each sequence, the analysis started after at least 1 second of stimulation to exclude the brief initial shortening of latency to the first 10 spikes. The temperature was measured on the anterior leg and kept above 30°C.

### Muscle fiber conduction velocity in situ

The MFCV in situ was tested by the first author (JK) with the same machine used for the needle EMG. We chose the TA muscle as it has extended and parallel fibers, which enable direct stimulation many centimeters away from the motor point. In another previous article, 16 we detailed the electrical activation technique to record the isolated muscle fiber action potentials (MFAPs) in the TA muscle and defined the reference values. In short, direct muscle fiber stimulation usually shows a polyphasic complex of many MFAPs with a very short rise time. The stimulation is delivered intramuscularly by a monopolar needle electrode, and the recording is by a CNE. Both needles are positioned at a right angle to the course of the muscle fibers, 16 at a fixed distance of 50 mm. The latency measurement for MFCV calculation was performed in the middle of the ascendent depolarizing line of the MFAPs ( Figure 1B ). The MFCV in situ was displayed in meters per second (50 mm/latency in ms). The MFAPs are confirmed when the MCD is below 5 μs in at least 10 (mostly more than 30) consecutive discharges, similar to the neuromuscular jitter. 17 The neuromuscular jitter (indirect muscle fiber stimulation) can be immediately discernible from the direct muscle fiber stimulation. The fastest to the slowest MFCV ratio (F/S ratio) was calculated. The temperature was measured on the anterior leg and kept above 30°C.

### Statistics

The descriptive statistics comprised the calculation of mean, median, standard deviation (SD), and percentile values for the continuous variables. The normality tests applied were as follows: Anderson–Darling, D'Agostino and Pearson, Shapiro-Wilk, and Kolmogorov-Smirnov (95% confidence interval, 95%CI). For the comparison of the variables, we used the parametric unpaired Student's t-test for normally distributed variables, the non-parametric Mann–Whitney U-test for non-normally distributed variables, and Z-score calculator for two population proportions. The comparison tests were set to a 95%CI. We used the Minitab Statistical Software (Minitab, LLC, State College, PA, United States).

### Data availability

The raw and anonymized data generated in the present study that support the conclusions will be made available by the authors without undue reservation upon reasonable request from qualified investigators.

## RESULTS

### Sample demographics and symptoms

The variables age, body mass index (BMI), MUP amplitude (µV), mean jitter, percentage of abnormal individual jitter values, MFCV (m/s), and F/S ratio for all groups are depicted in Table 1 . The mean interval between the SARS-CoV-2 infection and the interview and neurophysiological tests was of 11.9 (range: 6–24) months for the LC-no group and of 15.5 (range: 7–29) months for the LC-yes group.

**Table 1:** Variables analyzed in the study sample

Variables	Group	Mean	SD	Minimum	Median	Maximum
Age (years)	Controls	40.89	12.24	21	**40**	60
	LC-no	**36.56**	12.28	20	33	60
	LC-yes	**42.38**	14.12	24	42.50	68
Body mass index (kg/m ^2^ )	Controls	**27.32**	5.68	18.50	26	40.57
	LC-no	**27.61**	5.44	20.90	25.30	37.55
	LC-yes	**28.75**	6.32	18.83	28.45	41.21
Motor unit potential (µV)	Controls	1,920	607	1,256	**1,698**	3,438
	LC-no	1,699	540	1,129	**1,583**	3,391
	LC-yes	**1,813**	471	1,167	1,591	2,872
Mean jitter (abnormal: ≥ 26 µs)	Controls	**20.79**	2.48	15.82	21.09	25.13
	LC-no	**19.94**	3.13	14.42	19.71	26.32
	LC-yes	21.55	3.38	16.43	**21.75**	32.31
Percentage of individual jitter > 34 µs	Controls	4.78	4.22	0	**4.99**	16.66
	LC-no	**2.49**	3.94	0	0	13.33
	LC-yes	5	9.81	0	**1.67**	36.67
Muscle fiber conduction velocity (m/s)	Controls	**4.11**	0.37	3.33	4.06	5.05
	LC-no	**4.11**	0.25	3.71	4.14	4.69
	LC-yes	**4.12**	0.52	3.12	4.16	5.01
Muscle fiber conduction velocity: fast-to-slow ratio	Controls	1.73	0.49	1.27	**1.62**	3.74
	LC-no	**1.64**	0.22	1.24	1.65	2.03
	LC-yes	**1.82**	0.45	1.29	1.72	3.20

Abbreviations: LC, long coronavirus disease; SD, standard deviation.

Note: The numbers in bold represent parametric (mean) or non-parametric (median), according to the normality test of the variable sample.

The symptoms referred during the acute SARS-CoV-2 infection were tiredness (75%), gustation loss (68.8%), fatigue (62.5%), olfaction loss (59.4%), headache (56.3%), fever (46.9%), cough (43.8%), myalgia (43.8%), sore throat (37.5%), dyspnea (34.4%), rhinorrhea (28.1%), and diarrhea (25%). Tiredness or fatigue was acutely presented by 93.7% of the LC-yes group and by 62.5% of the LC-no group. The LC-yes group presented ≥ 6 symptoms during the SARS-CoV-2 infection in 68.8% of the cases, compared to 31.3% in the LC-no group. The Z-score calculator for 2 population proportions revealed a *p* value of 0.034, significant at *p* < 0.05. The SARS-CoV-2 infection was confirmed by PCR test in 87.5% and by antibodies in 12.5%.

Hypertension was referred in 2 controls and 4 COVID cases. One subject from the control group had undergone a BoNT injection in the facial muscles 3 years before the neurophysiological tests. We did not find any influence of those conditions on the mean jitter, MFVC, F/S ratio, or F-PEM score. Daily regular medications were used by 50% of the control group, 25% of the LC-no group, and 45.3% of the LC-yes group. We did not find any influence of those medicines on the mean jitter, MFVC, F/S ratio, or F-PEM score.

### Single-fiber electromyography

**Figure 2:**
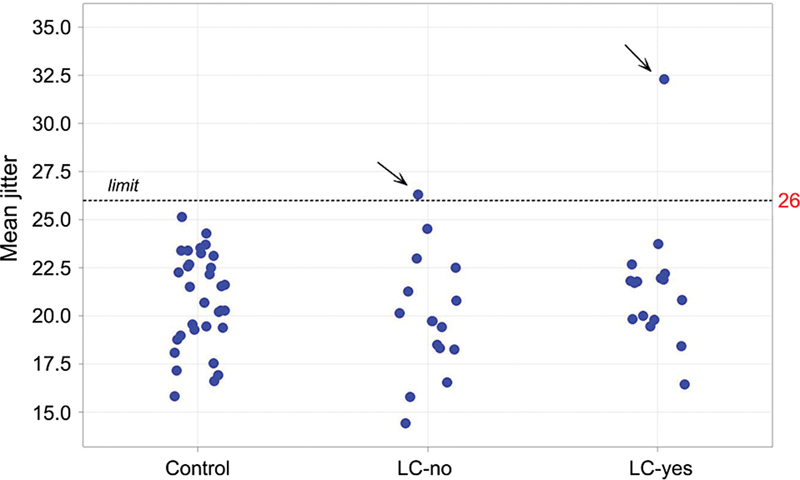
Plot with the mean jitter in all groups. Observe one abnormal jitter in the group without long coronavirus disease (LC-no) and another in the group with LC (LC-yes). Both groups presented acetylcholine receptor antibodies within the normal range, and none presented fluctuation weakness.

The mean jitter and the percentage of abnormal individual jitter values in the 3 groups are depicted in Table 1 and Figures 2 3 . One of the controls, one patient in the LC-no group, and two in the LC-yes group presented at least one abnormal jitter parameter; all of them have a negative acetylcholine receptor antibody (AChR-Ab).

**Figure 3:**
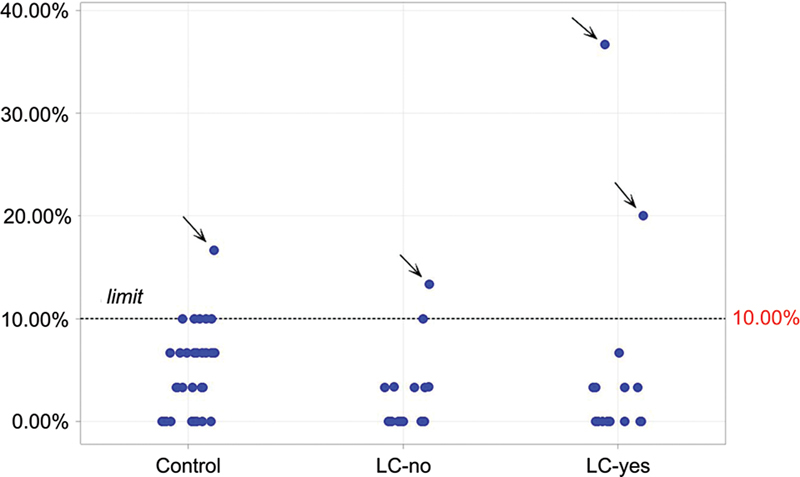
Plot with the abnormal individual jitter above 10% in all groups. Observe one abnormal percentage in the control group, one in the LC-no group, and two abnormal in the LC-yes group. All presented acetylcholine receptor antibodies within the normal range, and none presented fluctuation weakness.

### Muscle fiber conduction velocity and fast-to-slow ratio

**Figure 4:**
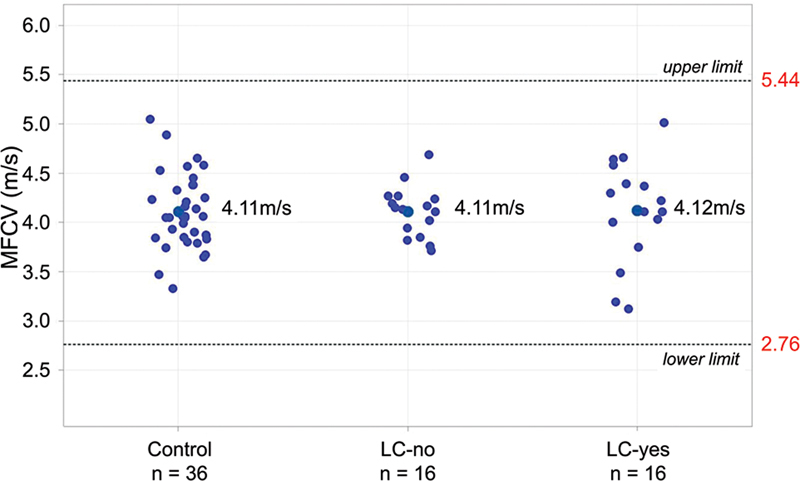
Plot with the muscle fiber conduction velocity in situ in all groups. Observe that none of the groups were outside the reference limits. None presented proximal fixed weakness nor abnormal motor unit potentials in needle electromyography.

The MFCV values and the F/S ratio are depicted in Table 1 and Figure 4 . The median values for the number of spikes for each MCD calculation were of 62, 56, and 36 for the control, LC-no, and LC-yes groups respectively. For each participant, the MFCV calculation was performed by a mean number of spikes of 21.8, 26.2, and 26.6 for the control, LC-no, and LC-yes groups respectively.

### Long COVID symptoms

**Figure 5:**
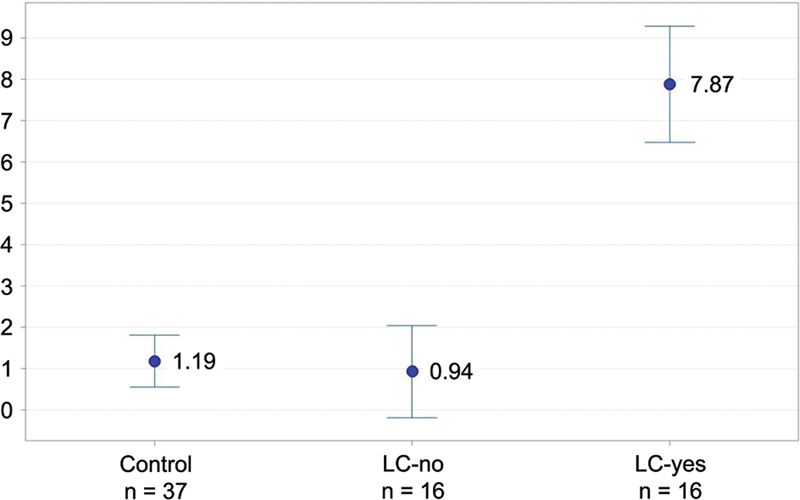
Plot with the mean fatigue/postexertional malaise score frequency in all groups (95% confidence interval for the mean). There are five questions and, for each of them, the scores are as follows: 0–without symptoms; 1–sometimes; 2–about half of the time; 3–most of the time; and 4–continuous.

The most reported residual symptom was F-PEM, which was referred by all patients in the LC-yes group, by 29.7% of the controls, and by 25.0% of the patients in the LC-no group. The *p* value was highly significant for the LC-yes group compared to the other groups. The usual answers were “intolerance to exercise,” “tired most of the time,” and “not achieving the same exercise schedule as before.” The F-PEM scores for each group were tabulated and compared based on the sum of points from the five questions outlined in the methods ( Figure 5 ).

### Comparison of the neurophysiological data

The comparison and significance of the mean jitter, the MFCV, and F-PEM frequency in the groups are depicted in Table 2 .

**Table 2:** Comparison of the mean jitter, MFCV, and F-PEM using the Mann-Whitney U-test

Group comparison	Variable	*p* value	Confidence	Significance
Controls versus LC-no	F-PEM	0.368	95.08%	No
Controls versus LC-yes	F-PEM	0	95.08%	High
LC-no versus LC-yes	F-PEM	0	95.21%	High
Controls versus LC-no	MFCV	0.774	95.15%	No
Controls versus LC-yes	MFCV	0.579	95.15%	No
LC-no versus LC-yes	MFCV	0.692	95.21%	No
Controls versus LC-no	Mean jitter	0.269	95.22%	No
Controls versus LC-yes	Mean jitter	0.768	95.22%	No
LC-no versus LC-yes	Mean jitter	0.147	95.21%	No

Abbreviations: F-PEM, fatigue/postexertional malaise; LC, long coronavirus disease; MFCV, muscle fiber conduction velocity.

## DISCUSSION

The rate of F-Pem in the LC-yes group was of 100%, and, in the LC-no group, of 25%. The mean sum of the points (0–4) of the 5 questions from the F-PEM scale was of 1.19, 0.94, and 7.94 for the control, LC-no, and LC-yes groups respectively. The frequency and severity of F-PEM reached a highly-significant difference ( *p* = 0.000) when the LC-yes group was compared to the other groups.

Overall, LC is found in 10 to 30% of the patients months after they have been infected with SARS-CoV-2, though the pooled global prevalence could be as high as 45.2%. 5 In some reports, 3 18 19 20 21 the incidence was estimated at 10 to 60.3% of non-hospitalized cases and 50 to 87% of hospitalized cases. The most common LC symptom is F-PEM, with prevalence rates ranging from 50% to 70% of patients, 6 7 8 9 10 substantially impacting the quality of life. 22 Fatigue/postexertional malaise reduces the ability of the muscle to produce maximum force during exercise. In the same way, regular daily activity is reduced by at least 50%.

The LC symptoms and their frequency have been described in many reports: F-PEM followed by cough, dyspnea, sleep disorders, adjustment disorders, and headache; 7 F-PEM, dyspnea, sleep disorder, and myalgia in 41%, 31%, 30%, and 22% of the patients respectively 23 ; F-PEM, anosmia/ageusia, dyspnea, difficulty concentrating, memory loss, and confusion; 24 F-PEM and dyspnea in 42%, followed by sleep disturbance in 28% of the patients; 25 F-PEM, sleep problems, headache, myalgia, arthralgia, and dyspnea; 26 F-PEM, muscle weakness, and myalgia; 27 28 and F-PEM in 33% and cognitive impairment in 20% of the patients. 18 In the present study, the 3 most frequent symptoms reported by SARS-CoV-2 patients during the acute infection were tiredness (75%), gustation loss (68.8%), and fatigue (62.5%). Unsurprisingly, tiredness and fatigue are directly related to F-PEM in LC, and at least one of those symptoms was present in the acute stage of the infection in 87.5% of the LC-yes compared to 37.5% in the LC-no group.

Certain risk factors have been linked to LC, such being an adult woman, 29 experiencing a long recovery period, persistent positivity on PCR after day 14 of the initial test, and severe acute COVID infection, such cases resulting in admission to an Intensive Care Unit (ICU) or requiring mechanical ventilation. 7 30 However, the presence of LC has no strict correlation with the severity of the acute stage of the illness or age. 5 24 31 32 In the current study, 68.8% of the LC-yes group presented ≥ 6 symptoms during the acute stage of the infection, compared to 31.3% of the LC-no group. Therefore, we can speculate that the presence of F-PEM and the number of symptoms during the acute stage of the SARS-CoV-2 infection could be risk factors for the development of LC.

During the acute stage of the SARS-CoV-2 infection, myalgia and fatigue have been described in 11 to 70% of the patients, and creatine kinase is elevated in 9% to 33%, 6 33 which could raise suspicion of acute muscle involvement. In a study, 11 the most common symptoms after hospital discharge from SARS-CoV-2 infection were fatigue or muscle weakness (63%). Due to the extremely high frequency of weakness, we can speculate if there was a reliable distinction between fatigue and weakness. In other reports, fatigue was found in 53.1% at 60 days 34 and in 52% at 10 weeks. 31

The cause of LC F-PEM symptoms is unknown. The pathophysiological mechanisms have been reported to include immune system abnormalities 35 and a possible association between elevated proinflammatory markers and F-PEM. 18 Nonetheless, LC has not been associated with routine laboratory markers of inflammation. 31 A postviral fatigue syndrome (PVFS) is claimed by some studies. 36 This condition was described decades ago after infectious mononucleosis (Epstein-Barr virus). Still, its relationship for triggering the symptoms was not subsequently confirmed. 37 Accordingly, the SARS-CoV-2 virus probably does not trigger PVFS. 3

We did not find statistically significant differences regarding the mean jitter, the percentage of abnormal individual jitter, the MFCV, and the F/S ratio values in the three groups, suggesting that these parameters do not play a role in LC cases with F-PEM. Accordingly, MUP morphology presented normal parameters in all patients. Out of 64 individuals across all groups, only 4 presented abnormal jitter parameters and were all negative for AChR-Ab. They may represent outlier findings—none presented with ptosis, diplopia, bulbar symptoms, or neck or limb fluctuating weakness. Neurogenic and myopathic MUP morphology that could increase the jitter, extend the muscle fiber conduction velocity range, and increase the F/S ratio was not found. Contrasting to our findings, Elanwar et al. 36 found a typical NMJ dysfunction with high jitter and abnormal decrement in 50% of their 46 LC cases. Traditionally, clinical fatigue scores have rarely been correlated with jitter parameter abnormalities. 38

Agergaard et al. 39 and Hejbøl et al. 6 found some muscle pathologic abnormalities and claimed it could cause F-PEM due to reduced energy supply. The hypothesis that myopathy could cause fatigue was also made due to quantitative electromyography. 6 Since inflammatory myopathy is a rare consequence of SARS-CoV-2 infection, millions of patients with LC and F-PEM may have an underlying etiology unrelated to muscle tissue damage. Agergaard et al. 39 found increased jitter, with impulse blocking in certain cases, in 17% and 25% of the LC patients in the TA and the extensor digitorum (ED) muscles respectively; some jitter recordings were taken from muscles with myopathic MUPs, introducing a bias toward jitter abnormality. Similar to our findings, the AChR-Ab was negative in all cases with increased jitter in another study by Agergaard et al. 40 Considering that about 20% of the patients with SARS-CoV-2 infection will develop LC, we could estimate about 154 million cases by June 2024. 2 Therefore, we can estimate that at least 77 million people will present F-PEM, and a true myopathy with proximal weakness in this plethora of cases did not occur.

The present study has certain limitations. Due to the time-consuming tests using needles, the small sample could interfere with some results. The negative results for the jitter parameters could be barely due to the sample size, as the comparison among the groups is insignificant. Most of the cases (87.5%) were confirmed by PCR. The antibodies (12.5%) were acceptable initially since other cases simultaneously occurred in the relatives. The MUP analysis was only performed in one muscle; however, we could exclude myopathy by adding to the MFCV F/S ratio and the clinical picture. Even though we included controls and patients using medicines, they were found to be in the same proportion in all groups; in fact, the LC-yes group used fewer medicines than the controls. We did not analyze the impact of vaccines on the main findings. However, aside from the anticipated mild fever or flu-like symptoms in the short term, none of the patients reported any long-term side effects.

In conclusion, F-PEM was found in all LC cases, and the electrophysiological findings did not indicate that muscle fiber or NMJ dysfunction are relevant factors in this condition.
